# Phenotypic Characterization of Female Carrier Mice Heterozygous for Tafazzin Deletion

**DOI:** 10.3390/biology12091238

**Published:** 2023-09-14

**Authors:** Michelle V. Tomczewski, John Z. Chan, Duaa M. Al-Majmaie, Ming Rong Liu, Alex D. Cocco, Ken D. Stark, Douglas Strathdee, Robin E. Duncan

**Affiliations:** 1 Department of Kinesiology and Health Sciences, Faculty of Health, University of Waterloo, 200 University Ave W., BMH1044, Waterloo, ON N2L 3G1, Canada; mvtomczewski@uwaterloo.ca (M.V.T.); l292zhan@uwaterloo.ca (J.Z.C.); almajmad@uoguelph.ca (D.M.A.-M.); mr2liu@uwaterloo.ca (M.R.L.); kstark@uwaterloo.ca (K.D.S.); 2 Transgenic Technology Laboratory, Cancer Research UK Beatson Institute, Switchback Road, Glasgow G61 1BD, Scotland, UK; d.strathdee@beatson.gla.ac.uk

**Keywords:** tafazzin, cardiolipin, Barth syndrome, mitochondria, energy metabolism, exercise capacity, mice, X-linked diseases, carriers, heterozygous

## Abstract

**Simple Summary:**

Barth syndrome (BTHS) is a disease that affects energy production. It results from mutations on the X chromosome, and thus it mainly affects males since they have only one copy (females have two X chromosomes). Although BTHS is rare, the consequences can be severe. BTHS affects the heart, immune system, and muscles, so boys and men with BTHS are at a high risk of heart attacks and death from infections, and they tire easily, which impacts quality of life. Women with one mutated X chromosome are typically thought to be asymptomatic, so they have not been studied very much. However, in other diseases caused by X chromosome mutations, females that were thought to be healthy developed symptoms after they got older. In this work, we studied female mice with one mutated X chromosome, which are models of female carriers of BTHS. We found that with advancing age, the carriers have some slowdown in their running speed, which is like that seen in male mice but less severe. They also weigh less and are better able to maintain their blood sugar, which could be beneficial. Our work shows that female carriers can have symptoms and supports further research in women.

**Abstract:**

Barth syndrome (BTHS) is caused by mutations in tafazzin resulting in deficits in cardiolipin remodeling that alter major metabolic processes. The tafazzin gene is encoded on the X chromosome, and therefore BTHS primarily affects males. Female carriers are typically considered asymptomatic, but age-related changes have been reported in female carriers of other X-linked disorders. Therefore, we examined the phenotype of female mice heterozygous for deletion of the tafazzin gene (*Taz*-HET) at 3 and 12 months of age. Food intakes, body masses, lean tissue and adipose depot weights, daily activity levels, metabolic measures, and exercise capacity were assessed. Age-related changes in mice resulted in small but significant genotype-specific differences in *Taz*-HET mice compared with their female *Wt* littermates. By 12 months, *Taz*-HET mice weighed less than *Wt* controls and had smaller gonadal, retroperitoneal, and brown adipose depots and liver and brain masses, despite similar food consumption. Daily movement, respiratory exchange ratio, and total energy expenditure did not vary significantly between the age-matched genotypes. *Taz*-HET mice displayed improved glucose tolerance and insulin sensitivity at 12 months compared with their *Wt* littermates but had evidence of slightly reduced exercise capacity. Tafazzin mRNA levels were significantly reduced in the cardiac muscle of 12-month-old *Taz*-HET mice, which was associated with minor but significant alterations in the heart cardiolipin profile. This work is the first to report the characterization of a model of female carriers of heterozygous tafazzin deficiency and suggests that additional study, particularly with advancing age, is warranted.

## 1. Introduction

Cardiolipin is a component of the inner mitochondrial membrane, constituting approximately 15–20% of the glycerolipids present [1]. The dimeric nature of cardiolipin makes it structurally unique among phospholipids, allowing for esterification of four fatty acyl chains rather than two [2]. While this permits, in theory, the generation of thousands of different possible molecular species of cardiolipin, lipidomic analyses indicate that only a handful of major species predominate [3]. In general, cardiolipin species in all tissues are enriched with 18-carbon fatty acyl chains, and while different tissues tend to have different cardiolipin molecular profiles, there is often conservation in these tissue profiles across organisms [2]. One molecular species, tetralinoleoyl (L4)-cardiolipin), which is comprised of four linoleic acid moieties, is particularly enriched in heart muscle from various organisms, ranging from approximately 80% of cardiolipin molecules in human hearts [4] to 1/3rd of cardiolipin molecules in mouse hearts [5].

Cardiolipin synthesis occurs initially in the Kennedy pathway [6]. However, the enzymes involved in de novo synthesis pathways lack the acyl-chain specificity needed to create the signature molecular profiles that are evident in various tissues [2]. To this end, at least five cardiolipin remodeling enzymes have been identified [7,8,9,10,11,12]. These enzymes function to re-esterify fatty acyl chains onto monolyso- or dilyso-cardiolipin produced from the action of various phospholipases on nascent cardiolipin, resulting in the generation of a mature form of this complex lipid [2]. Among these, the enzyme tafazzin has been extensively studied as a phosphatidylcholine:monolysocardiolipin transacylase that is critical for the generation of L4-cardiolipin [2]. 

Tafazzin is highly expressed in cardiac and skeletal muscle but is also expressed at variable levels in other tissues [2]. Mutations in tafazzin cause Barth syndrome (BTHS), a disease associated with L4-cardiolipin deficiency, resulting in variable clinical findings including cardiac and skeletal myopathies as well as immunological deficits and changes in cognition, taste, and sensory perception, among other symptoms [13]. BTHS is an X-linked recessive disorder, and therefore individuals with Barth Syndrome are almost entirely male [14]. The prevalence of the disease is low, affecting an estimated 1:300,000 to 1:400,000 live births [14]. The number of female carriers likely exceeds the number of clinically diagnosed males, however, since historically 70% of affected males perished in childhood due to the disease [15,16]. 

Female carriers of tafazzin mutations are typically considered clinically asymptomatic [14], but whether heterozygous tafazzin deficiency causes phenotypic changes at either younger or older ages in female carriers remains poorly understood. In other X-linked disorders of lipid metabolism, such as X-linked adrenoleukodystrophy, clinically relevant symptoms have been reported with advancing age [17]. We have therefore studied female mice heterozygous for tafazzin at ages 3 and 12 months.

## 2. Materials and Methods

### 2.1. Mice

The generation and maintenance of the *Taz*-KO mice were approved by a UK Home Office Licence (PP9886217). Animal procedures, including breeding and all experiments performed at the University of Waterloo (UW), were approved by the UW Animal Care Committee under AUPP#30055 (17-19), approved 25 July 2017; AUPP#41822, approved 27 August 2020; and AUPP#43431, approved 9 July 2021, and comply with Canadian Council on Animal Care guidelines and ARRIVE guidelines. Mice were housed in a temperature- and humidity-controlled environment on a 12:12 h light/dark cycle with enrichment materials and free access to standard rodent chow (Teklad 22/5 Rodent diet from Envigo, Haslett, MI, USA) and water, together with their sex-matched littermates.

Gene targeting was performed at the Beatson Institute (Cancer Research UK), resulting in the generation of mice with a germline deletion of exons 5–10 of tafazzin, and has been previously reported in detail [18,19]. Mice were backcrossed onto a C57BL/6J line >20 generations before use. Heterozygous females (*Taz*^Δ/+^ [*Taz*-HET]) and wildtype (*Wt*) littermate females (*Taz*^+/+^) used in experiments were generated by mating male C57BL/6J mice (Jackson Labs, Bar Harbor, ME, USA) with *Taz*^Δ/+^ females. This also generated *Wt* male (*Taz*^+/Y^) and hemizygous null (*Taz*^Δ/Y^ [*Taz*-KO]) male mice. Their phenotypic characterization has been reported separately [18,20]. 

Genotype was analyzed using an extract of genomic DNA isolated from ear punches, and the presence of the KO *Taz* allele was verified by generation of a 280 bp amplicon, visualized under ultraviolet light following gel electrophoresis using 1% TAE-agarose gels with ethidium bromide. The amplicon was produced by PCR using FastStart^TM^ Master Mix (Roche Life Science, Mississauga, ON, Canada) and a Bio-Rad^®^ T100 thermocycler (Mississauga, ON, Canada) under the following conditions: 95 °C for 4 min; 39 cycles of 95 °C for 30 s, 56 °C for 30 s, 72 °C for 1 min; and a final extension step of 72 °C for 7 min, with the KO-U1 (5′-CCAAGTTGCTAGCCCACAAG-3′) forward primer and WT-D1 (5′-CAGGCACATGGTCCTGTTTC-3′) reverse primer. Since the mating strategy could only produce *Wt* (*Taz*^+/+^) or heterozygous (*Taz*^Δ/+^) females, detection of the null allele was sufficient to genotype mice as heterozygous, with those lacking the null allele designated as *Wt.*

### 2.2. Comprehensive Laboratory Animal Monitoring System Measures

Indirect calorimetry measures were analyzed in 3- and 12-month-old mice over a 24 h period, essentially as we have previously described [20]. Twenty-four hours prior to testing, mice were single-housed to allow for determination of food consumption and to assist them in adjusting to individual housing conditions prior to entering the Comprehensive Laboratory Animal Monitoring System (CLAMS, Columbus Instruments, Columbus, OH, USA). Mice were then placed in individual CLAMS chambers for an additional 2 h of acclimatization prior to the onset of data recording. Throughout the experiment, mice had unlimited access to chow and water and were maintained at room temperature (22–23 °C) with 12:12 h light/dark cycle (light 07:00–19:00 h, dark 19:00–07:00 h). Air flow in the chambers was exchanged at 0.5 L/min. Immediately prior to testing, gas sensors were calibrated with mixtures of standard gases (20.5% oxygen, 0.5% carbon dioxide, and nitrogen at balance), and oxygen consumption (VO_2_; mL/kg/h) and carbon dioxide production (VCO_2_; mL/kg/h) rates were analyzed at 28-min intervals. The respiratory exchange ratio (RER) was calculated from measures of VCO_2_/VO_2_ [21]. The Lusk equation [(3.815 + 1.232 × RER) × VO_2_ (in liters)] was used to calculate total energy expenditure (TEE), which was normalized to individual body weights (kg) [22]. Infrared beam breaks were used to determine activity in three directions: x (locomotion), y (ambulation), and z (rearing) planes. 

### 2.3. Glucose Tolerance Testing (GTT)

Mice at 12 months of age were tested for glucose tolerance, as we have previously described [20]. Food was withdrawn 6 h prior to testing at 15:00 h. Tail vein blood glucose levels were measured at baseline and 15, 30, 60, 90, and 120 min following *i*.*p*. injection of D-glucose (2.0 g/kg) using Freestyle Lite test strips and glucometer (Abbot Laboratories, Mississauga, ON, Canada). Area-under-the-curve (AUC) analysis was performed to allow for comparisons between overall test effects.

### 2.4. Insulin Tolerance Testing (ITT)

Twelve-month-old mice were tested for insulin sensitivity, essentially as we have previously described [20]. Following a 2 h fast (13:00–15:00 h), baseline glucose levels were measured using the Freestyle Lite glucose monitoring system, and then mice received an *i*.*p*. injection of insulin (0.5 U per kg body weight). Glucose levels were monitored in tail-vein blood at 15, 30, 60, 90, and 120 min and analyzed to determine AUC responses [23].

### 2.5. Treadmill Exercise Capacity Test

Mice at 3 and 12 months of age were subjected to an incremental treadmill exercise test using a five-lane motor-driven treadmill (Panlab; Harvard Apparatus, Barcelona, Spain) with a fixed slope of 5°, based on prior protocols [24,25], essentially as we have previously described [20]. Briefly, on the first 3 days of training, mice were placed on the treadmill in an immobile setting for 5 min, and then the treadmill was turned on to settings of 5 cm/s for a period of 5 min, followed by increases to 10 cm/s for 2 min, and then 15 cm/s for 3 min. On the third day, mice were placed on the static treadmill for 5 min, then it was set to speeds of 5 cm/s for a 3 min interval, 10 cm/s for 2 min, 15 cm/s for 2 min, and then 20 cm/s for 3 min. Mice rested on Day 4. An exhaustive exercise test was performed on Day 5 by placing mice on the treadmill for 5 min at a stationary setting, followed by an initial speed of 10 cm/s, which was increased by 3 cm/s every 2 min until a maximum speed of 70 cm/s was achieved. Running time and total distance ran until exhaustion were monitored. Sessions were performed from 16:00–19:00 h, which fell within the dark photoperiod for the mice since mice are normally most active nocturnally. Sessions were completed in a dark room, where illumination was provided using red lighting to prevent disruption to the dark cycle.

### 2.6. Tafazzin Gene Expression

Total RNA was extracted from mouse heart tissue using TRIzol^®^ Reagent (Invitrogen, Waltham, MA, USA) according to the manufacturer’s protocol after tissue was mechanically disrupted using a POLYTRON^®^ PT 1200 E homogenizer (VWR, Radnor, PA, USA) set at maximum speed. Quantity of RNA was measured spectrophotometrically using a Nanodrop 2000 Spectrophotometer (ThermoFisher, Waltham, MA, USA), and 2 μg of RNA per 20 μL reaction was used to synthesize cDNA via random hexamer priming using a High-Capacity cDNA Reverse Transcription kit from Applied Biosystems (Waltham, MA, USA) according to the manufacturer’s protocol. cDNA was diluted 1:5 in ddH_2_O. The quantitative real-time (qRT)-PCR duplex reactions contained 7.5 μL PerfeCTa ToughMix qPCR MasterMix (Quantabio, Beverly, MA, USA), 0.75 μL each of the Taqman gene expression assays for FAM-labeled tafazzin (Mm04239390_g1), spanning exons 2/3, and VIC-labeled β-actin (Mm02619580_g1) (Applied Biosystems, Mississauga, ON, Canada), 5 μL of ddH_2_O, and 1 μL of cDNA. Transcript levels were measured on a CFX-96 Connect Real Time qPCR Detection System (BioRad, Hercules, CA, USA). Thermal cycling conditions were 50 °C for 2 min, then 95 °C for 20 s, followed by 40 cycles at 95 °C for 3 s, then 60 °C for 30 s. All measurements were run in duplicate. Tafazzin expression was analyzed using the ΔΔCt method, with the Ct values normalized to β-actin.

### 2.7. Gas Chromatography Analysis of Cardiolipin

Mouse heart tissue was homogenized in phosphate-buffer saline (PBS) using a TissueLyser II for 2 min at a frequency of 25 Hz. Total lipids were extracted from the homogenate by the addition of 2:1 (*v*/*v*) chloroform:methanol with the antioxidant butylated hydroxytoluene (BHT) and overnight storage. The next day, phosphate buffered saline was added, the samples were vortexed, and then centrifugated at 3000 rpm for 5 min to separate the organic and aqueous phases. The organic phase was carefully collected and desiccated under a stream of nitrogen gas. To separate the individual phospholipid species, the organic phase was resuspended in chloroform and applied onto Silica Gel HF plates measuring 20 cm × 20 cm with a layer thickness of 250 μM (Analtech Inc., Cole-Parmer Canada, Montreal, QC, Canada), and resolved using thin-layer chromatography with a solvent system of chloroform:methanol:2-propanol:0.25% KCl:trimethylamine (30:9:25:6:18, *v*/*v/v*/*v/v*) [26]. Cardiolipin bands were detected using UV illumination after spraying with 0.1% 2,7-dichlorofuorescin in methanol (*w*/*v*) and scrapped in reference to known cardiolipin standards (Avanti Polar Lipids, Millipore Sigma, Mississauga, ON, Canada). To prepare for gas chromatography (GC) analysis, the fatty acyl species within cardiolipin underwent transesterification to fatty acyl methyl esters on a heating block for 1 h at 95 °C using 14% boron trifluoride in methanol and hexane (Thermo Scientific, Bellfonte, PA, USA), overlaid with nonadecanoic acid (19:0) as an internal standard (Nu-Chek Prep, Elysian, MN, USA). Following centrifugation at 3000 rpm for 5 min, the hexane layer was collected, transferred, desiccated under nitrogen gas, and resuspended in 65 μL of heptane. Subsequent analysis using gas chromatography with flame ionization detection was performed using a SCION 8300 GC (Scion Instruments Canada, Edmonton, AB, Canada) equipped with a DB-FFAP 15 m length × 0.10 mm inner diameter × 0.10 μm film thickness nitroterephthalic acid-modified polyethylene glycol capillary column (J&W Scientific/Agilent Technologies, Mississauga, ON, Canada) with hydrogen as the carrier gas. Specifically, 1 μL of samples were introduced by an Scion 8400Pro autosampler into the injector and heated to a temperature of 250 °C with a split ratio of 100:1. The initial temperature was set at 150 °C and maintained for 0.25 min, followed by a ramp of 35 °C/min to 200 °C, a 1 °C/min ramp to 211 °C, and an 80 °C/min ramp to 245 °C with a final hold of 4 min. The flame ionization detector temperature was maintained at 300 °C, while the make-up gas comprised air and nitrogen, flowing at rates of 300 and 30 mL/min, respectively. Sampling frequency was set at 50 Hz. The quantification of fatty acyl composition was expressed both in terms of concentrations (μg fatty acids per mg of tissue) and relative weight percentages, reflecting the proportion of each fatty acyl species within the total mass of fatty acids analyzed. Subsequently, the total content of cardiolipin was computed based on the cumulative mass of all cardiolipin fatty acyl species present within each sample.

### 2.8. Statistical Analyses

Comparisons between *Wt* and *Taz*-HET mice at 3 and 12 months of age were conducted using a two-way ANOVA to detect an interaction between genotype and age as main factors. Following identification of significant effects, Holm-Sidak’s multiple comparison tests were used to identify significant differences in means within genotypes between ages and significant differences between age-matched animals with different genotypes.

Blood glucose levels across timepoints were compared between *Wt* and *Taz*-HET mice by repeated measures ANOVA. Area-under-the-curve (AUC) analyses were performed to determine the overall response of individual mice, and means from each group were compared using Student’s t-test.

Differences between genotypes for tafazzin gene expression and cardiolipin measures were assessed by Student’s t-test. 

GraphPad Prism (version 9.0.0 for Mac, San Diego, CA, USA) was used to perform all statistical analyses. Data are presented as means ± standard error of the mean (SEM), and differences were considered significant when *p* < 0.05. 

## 3. Results

### 3.1. Body, Organ, and Lean Tissue Weights

**Figure 1:**
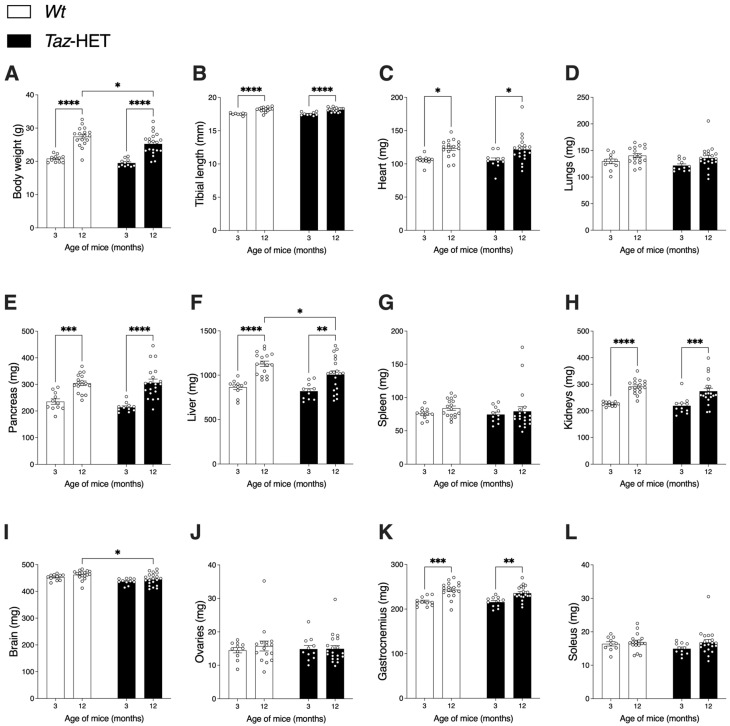
Body weights and lean tissue masses obtained by dissection of female *Wt* and *Taz*-HET mice at 3 and 12 months of age. Body weights (**A**) and tibial lengths (**B**), organ weights (**C**–**J**), and skeletal muscle masses (**K**,**L**) are shown according to genotype and age. Data are means ± SEM, with individual datapoints shown as circles, *n* = 11 per group at 3 months of age and *n* = 17–20 per genotype at 12 months of age. Comparisons between genotypes for age-matched mice, and comparisons between ages for mice within genotype groups are shown: * *p* < 0.05, ** *p* < 0.01, *** *p* < 0.001, **** *p* < 0.0001.

Mice of both genotypes grew from 3 to 12 months of age, as evidenced both by increases in absolute body weights (Figure 1A) and increases in tibial lengths within genotype groups (Figure 1B). At 3 months of age, there were no significant differences in body weights between *Taz*-HET and *Wt* mice (19.50 ± 1.13 g vs. 20.83 ± 1.10 g, respectively), but by 12 months, *Taz*-HET mice weighed 8% less than their *Wt* littermate controls (25.25 ± 3.24 g vs. 27.46 ± 2.90 g, respectively) (Figure 1A). Tibial lengths, however, did not differ between age-matched *Wt* and *Taz*-HET mice at either age examined, suggesting that differences in body weights at 12 months may reflect differences in body composition rather than absolute body stature per se. 

Masses of hearts (Figure 1C), pancreas (Figure 1E), kidneys (Figure 1H), and gastrocnemius muscles (Figure 1K) increased with age in both genetic groups of mice but did not differ between age-matched genotypes. Masses of lungs (Figure 1D), spleens (Figure 1G), ovaries (Figure 1J), and soleus muscles (Figure 1L) did not differ significantly between genotypes at either age examined or between ages within a genotype group, indicating that maximal size for these organs and tissues was already attained by 3 months in all animals. 

Only two organs differed in size between genotype groups, and significant differences were evident only at 12 months of age. Liver weights increased significantly from 3 to 12 months of age in both *Wt* and *Taz*-HET mice (Figure 1F). However, at 12 months of age, *Taz*-HET liver weights were ~11% lower than *Wt* liver weights. 

Brain masses did not change significantly from 3 to 12 months of age in either the *Wt* or *Taz*-HET mouse groups. At both 3 and 12 months of age, brain masses were ~4% lower in *Taz*-HET mice compared with age-matched *Wt* mice, but this was only statistically significantly different at 12 months of age (Figure 1I), possibly due to the greater power associated with the larger sample size examined at this timepoint.

### 3.2. Adipose Tissue Masses

**Figure 2:**
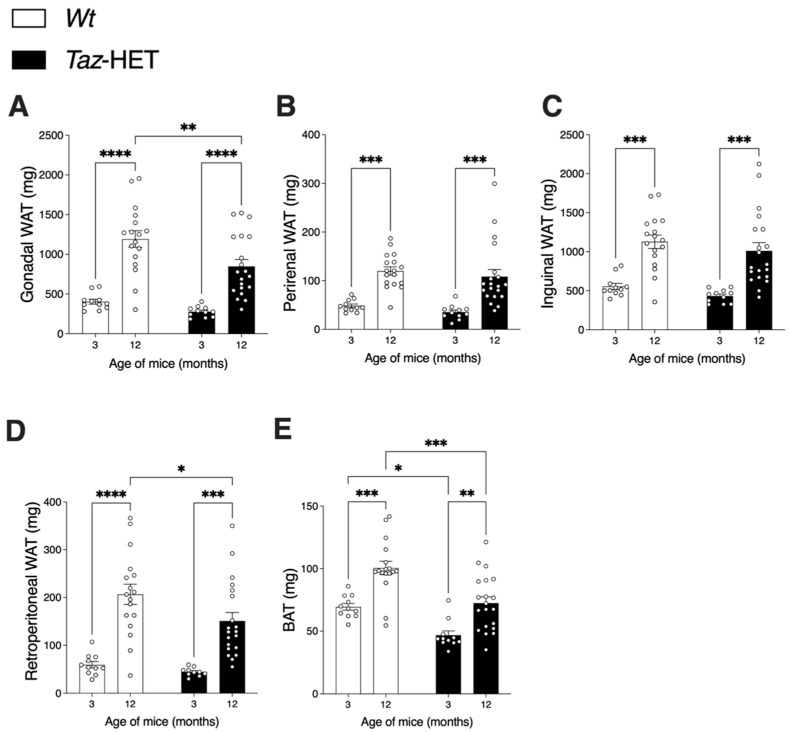
White (WAT) and brown adipose tissue (BAT) masses obtained by dissection from female *Wt* and *Taz*-HET mice at 3 and 12 months of age. WAT depots (**A**–**D**) and BAT depots (**E**) are shown according to genotype and age. Data are means ± SEM, with individual datapoints shown as circles, *n* = 11 per group at 3 months of age and *n* = 17–20 per genotype at 12 months of age. Differences were analyzed by 2-way ANOVA, and comparisons between genotypes for age-matched mice and between ages for mice within genotype groups are shown: * *p* < 0.05, ** *p* < 0.01, *** *p* < 0.001, **** *p* < 0.0001.

From 3 to 12 months of age, both *Wt* and *Taz*-HET mouse white adipose tissue (WAT) visceral (i.e., gonadal, perirenal, and retroperitoneal) and subcutaneous (i.e., inguinal) depots increased significantly in mass (Figure 2). At 3 months, there were no significant differences between *Wt* and *Taz*-HET mice in the mass of any individual WAT depot (Figure 2A–D). At 12 months, however, gonadal (Figure 2A) and retroperitoneal (Figure 2D) WAT depots were, respectively, 29% and 31% lower in mass in *Taz*-HET compared with *Wt* mice (Figure 2A,D).

Interscapular brown adipose tissue (BAT) depots increased in mass from 3 to 12 months of age in both *Wt* and *Taz*-HET mice (Figure 2E). *Taz*-HET mice had 33% smaller BAT masses at 3 months and 28% smaller BAT masses at 12 months compared with their age-matched *Wt* littermates.

### 3.3. Food Intake

**Figure 3:**
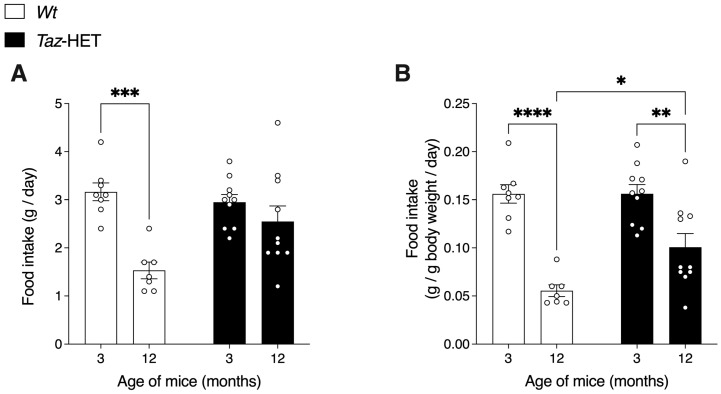
Total and weight-normalized daily food intakes of cohorts of 3- and 12-month-old mice. Twenty-four h absolute food intake (**A**) and food intake normalized to body weight (**B**) are shown. Data are means ± SEM, with individual datapoints shown (circles), *n* = 7–8 (*Wt*), *n* = 10 (*Taz*-HET), * *p* < 0.05, ** *p* < 0.01, *** *p* < 0.001, **** *p* < 0.0001.

Using the CLAMS, a series of measures were performed. Mouse food intake was averaged over a 24 h period (Figure 3A) and also normalized to body weight (Figure 3B). At three months of age, absolute and weight-normalized food intake did not differ between genotypes. Total and weight-normalized food intakes decreased in *Wt* mice from 3 to 12 months. During this same period, total food intakes did not decrease significantly in *Taz*-HET mice, although there was a significant decline in weight-normalized measures. Food intake measured per gram of body weight at 12 months of age was higher in *Taz*-HET mice compared with their *Wt* littermates.

### 3.4. Movement

**Figure 4:**
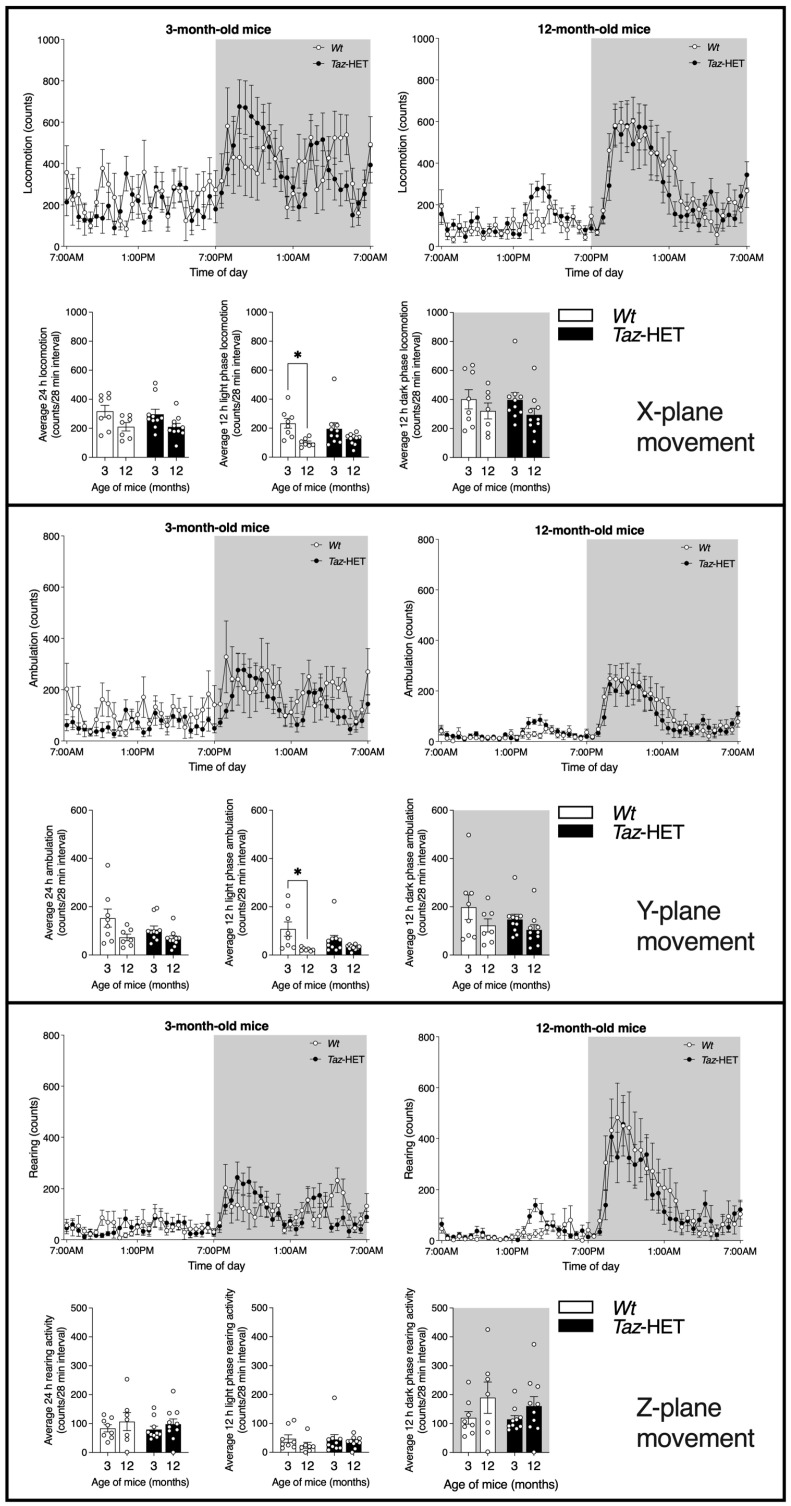
Movement of 3- and 12-month-old cohorts of mice over a period of 24 h housed in metabolic chambers equipped with infrared beams. Corresponding bar graphs of the daily and photoperiod average number of beam breaks per 28-min interval are shown. The movements of mice in the X (locomotor) plane (upper panel), Y (ambulatory) plane (middle panel), and Z (rearing) plane (lower panel) are shown. Shading indicates the duration of the dark cycle. Data are means ± SEM, with individual datapoints shown (circles), *n* = 7–10, * *p* < 0.05.

Mice at 3 and 12 months of age were monitored in the CLAMS apparatus, with motion recorded over a 24 h period. Motion was detected through the monitoring of infrared beam breaks. The locomotion axis records breaks of the X-plane, which include all beam breaks due to combined repetitive activity such as feeding or grooming, in addition to activity from walking, and is termed locomotor activity. The Y-plane runs the length of the enclosure and records instances when a mouse breaks two different beams, indicating a traverse of the cage, which is termed ambulatory activity. Monitoring of the rearing axis only accounts for the infrared beam breaks in the Z-plane, such as when mice rear up on their hind legs (Figure 4). 

Locomotor activity is shown in Figure 4 (upper panel), where line graphs show total beam breaks across the X-plane of the cage during each 28 min interval for 3-month-old (top, left) and 12-month-old (top, right) *Taz*-HET mice and their *Wt* littermates. Analysis of the average daily locomotor activity per 28 min interval indicated no significant differences between groups across ages and genotypes (bottom, left). *Wt* mice experienced an age-related significant decline in locomotor activity during the light phase, when mice are typically less active (bottom, middle), but otherwise there were no significant differences in average 28 min interval beam breaks across the X-plane in either the light or dark (bottom, right) cycles.

Line graphs of ambulatory activity (Figure 4, middle panel, top) indicate total values during 28 min intervals for beam breaks across the Y-plane of the cage recorded over a 24 h period, including both the light and dark cycles. Averages for these 28 min intervals are shown in this panel for the full 24 h period (bottom, left), the 12 h light phase (bottom, middle), and the 12 h dark phase (bottom, right). Similar to results seen for locomotor activity, ambulatory activity decreased significantly from 3 to 12 months of age in *Wt* mice during the light phase, specifically, but there were no significant differences between genotypes in age-matched mice and no other age-related differences within genotype groups. 

Rearing activity, denoted by activity in the Z-plane, was recorded during a 24 h period, and the total number of rearing events per 28 min interval is plotted according to genotype and age of mice (Figure 4, lower panel, top). Daily averages of these values (bottom, left) and averages during the light (bottom, middle) and dark (bottom, right) phases indicated no significant effects of age or genotype.

### 3.5. Respiratory Exchange Ratio and Total Energy Expenditure

**Figure 5:**
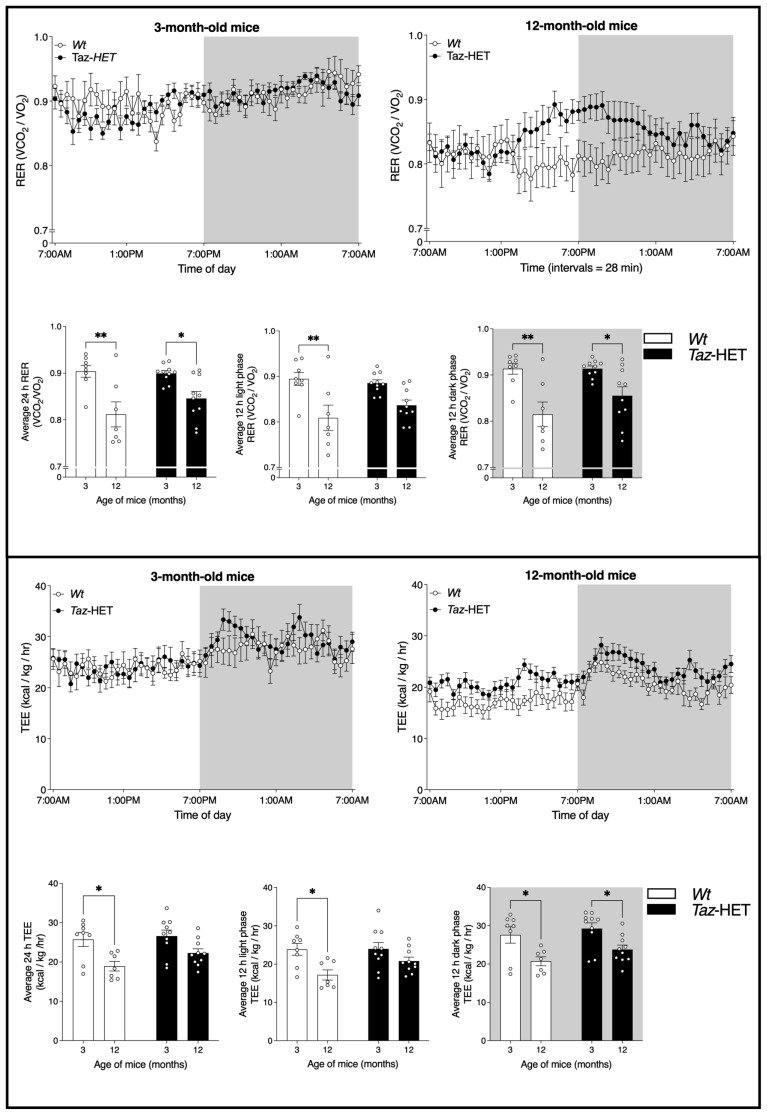
Respiratory exchange ratio (RER) and total energy expenditure (TEE). Cohorts of mice were monitored at 3 and 12 months of age for 24 h in the CLAMS apparatus, with measures taken every 28 min. Corresponding bar graphs of the daily and photoperiod average RER and TEE are shown. Data are means ± SEM, with individual datapoints shown (circles), *n* = 7–10, * *p* < 0.05, ** *p* < 0.01.

Respiratory exchange ratios (RER) and total energy expenditures (TEE) were analyzed for 3- and 12-month-old *Wt* and *Taz*-HET mice and recorded every 28 min (Figure 5, top and bottom panels).

Line graphs show comparisons of RER measures taken every 28 min from *Wt* and *Taz*-HET mice at 3 months (Figure 5, upper panel, top right) and 12 months (top left) of age. Both *Wt* and *Taz*-HET mice underwent an age-related decrease in 24 h and dark phase RER (Figure 5, top panel, lower left and right). During the light phase, RER declined significantly from 3 to 12 months of age for *Wt* but not *Taz*-HET mice (Figure 5, top panel, middle). No significant differences were observed between age-matched *Wt* and *Taz*-HET mice in RER. 

TEE measures are shown in Figure 5 (lower panel), with line graphs illustrating measures at 28 min intervals in 3-month-old (top, left) and 12-month-old (top, right) mice. Average TEE decreased significantly from 3 to 12 months of age in *Wt* mice during both the light and dark phases and also over the 24 h period measured overall (Figure 5, lower panel, bottom left, middle, and right). *Taz*-HET mice, however, only exhibited a statistically significant age-related decrease in TEE during the dark phase (bottom, right). Age-matched mice did not differ significantly in TEE measures by genotype. 

### 3.6. VO_2_ and VCO_2_

**Figure 6:**
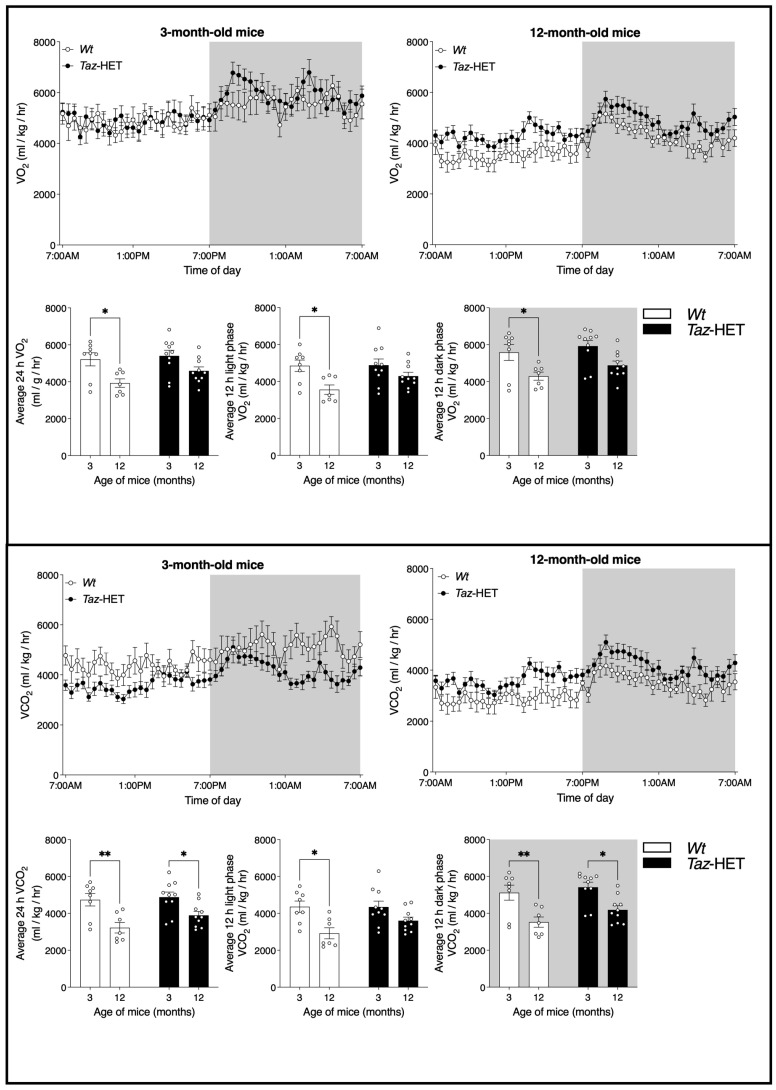
VO_2_ and VCO_2_ rates in 3- and 12-month-old cohorts of *Wt* and *Taz*-HET mice. Three- and twelve-month-old mice were monitored in the CLAMS apparatus, where measures of oxygen consumption rate and carbon dioxide production rate were calculated every 28 min. Corresponding bar graphs of the daily and photoperiod averages of VO_2_ and VCO_2_ are shown. Data are means ± SEM, with individual datapoints shown (circles), *n* = 7–10, * *p* < 0.05, ** *p* < 0.01.

Oxygen consumption rates (VO_2_) were analyzed in 3- and 12-month-old *Wt* and *Taz*-HET mice. Line graphs depicting average VO_2_ measures taken over 28 min intervals are shown (Figure 6, upper panel, top left and right). *Wt* mice exhibited a significant decrease in oxygen consumption with aging (upper panel, bottom left, middle, and right). Notably, this decrease in VO_2_ was evident when averages over the entire 24 h period were considered, as well as when averages were compared during the less active light period and the more active dark period. In *Taz*-HET mice, however, oxygen consumption rates did not decline significantly from 3 to 12 months of age, suggesting greater resistance to age-related metabolic changes. In age-matched mice, however, there were no significant differences between *Wt* and *Taz*-HET littermates in measures of VO_2_. 

Carbon dioxide production rates (VCO_2_) were also analyzed over a 24 h period in 3- and 12-month-old littermates. VCO_2_ rates were graphed at 28 min intervals for 3-month-old (Figure 6, lower panel, top right) and 12-month-old (Figure 6, lower panel, top left) *Wt* and *Taz*-HET mice. Calculation and analysis of average rates of carbon dioxide production indicated significant age-related decreases in both *Wt* and *Taz*-HET mice from 3 to 12 months of age overall (Figure 6, lower panel, bottom left) and specifically during the 12 h dark period (bottom right). During the light period, an age-related decrease in VCO_2_ was evident in *Wt* mice but not in *Taz*-HET mice (bottom middle). As with measures of VO_2_, there were no significant differences between age-matched mice of different genotypes in VCO_2_ overall or during either the light or dark periods. 

### 3.7. Glucose Tolerance and Insulin Sensitivity

Genotype-specific differences in body weights and WAT masses were observed in older but not younger mice (Figure 1 and Figure 2), although both genotypes undergo a decline in respiratory exchange ratio with age, suggesting changes in the relative use of major fuel types. Thus, tests of glucose tolerance (GTT) and insulin sensitivity (ITT) were performed in 12-month-old mice to determine if older animals exhibit genotype-specific differences. 

**Figure 7:**
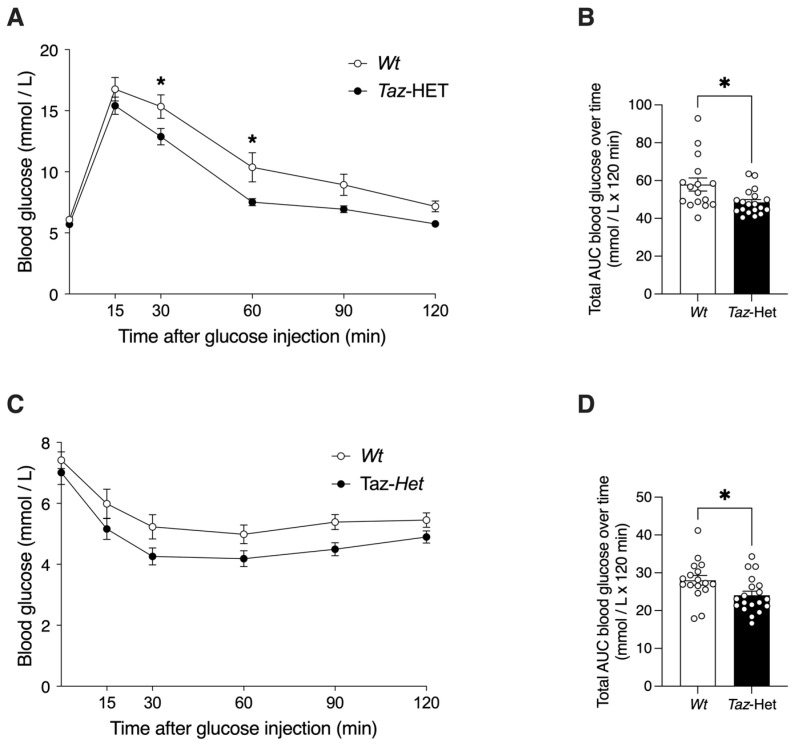
Blood glucose responses in 12-month-old female *Wt* and *Taz*-HET mice during GTT and ITT. Blood glucose responses immediately prior to *i*.*p*. injection with glucose and at 15, 30, 60, 90, and 120 min after injection were determined (**A**), and total glucose excursions were calculated by area-under-the-curve (AUC) analysis (**B**). Following *i*.*p*. insulin injection, blood glucose responses were monitored at the timepoints indicated (**C**), and total insulin-mediated glucose excursions were calculated by AUC analysis and mean AUC values during the experiment were compared (**D**). Data are means ± SEM, with individual datapoints shown (circles), *n* = 16–19, * *p* < 0.05, with differences indicated between mice of different genotypes at the same timepoint (**A**,**C**) or as indicated on the graph (**B**,**D**).

There were no significant differences in baseline glucose measures between *Wt* and *Taz*-HET mice (Figure 7A). Following injection of mice with glucose, both genotypes demonstrated a rise in blood glucose by 15 min, although mean levels did not differ significantly between genotypes at that timepoint. Blood glucose levels were, on average, 2.41 mM lower in *Taz*-HET mice compared with their *Wt* littermates at 30 min post-injection and 2.74 mM lower at 60 min. Total blood glucose excursions were quantified by calculation of total AUC (Figure 7B) and were 16% lower in 12-month-old *Taz*-HET mice compared with *Wt* mice, indicating significantly better glucose disposal following an injected glucose challenge.

Blood glucose concentrations at the start of the ITT protocol did not differ significantly between groups or at specific timepoints post-injection when analyzed by repeated measures ANOVA (Figure 7C). However, measures of total AUC indicated that blood glucose levels were suppressed to a greater overall extent in *Taz*-HET mice compared with their *Wt* littermates, indicating a greater response to insulin injection.

### 3.8. Exercise Endurance Capacity

**Figure 8:**
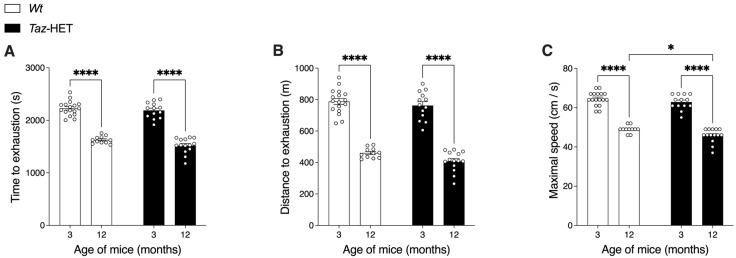
Treadmill exercise capacity testing. Three- and twelve-month-old cohorts of *Wt* and *Taz*-HET mice were trained for 3 days and then tested after one day of rest for maximal exercise capacity, measuring time to exhaustion (**A**), distance to exhaustion (**B**), and maximal speed achieved (**C**). Data are means ± SEM, with individual datapoints shown (circles), *n* = 11–17, * *p* < 0.05, **** *p* < 0.0001.

The exercise capacity of mice was determined using an exhaustive treadmill running protocol after 3 days of training. Three measures of exercise performance were determined. The time to exhaustion declined significantly in both *Wt* and *Taz*-HET mice from 3 to 12 months of age (Figure 8A). However, there was no significant difference in time to exhaustion between the genotypes when mice were compared at the same ages. Furthermore, regardless of genotype, mice at 3 months of age were able to run over 1/3 longer than mice at 12 months of age. 

Similar effects were observed for distance to exhaustion (Figure 8B). Twelve-month-old mice of either genotype achieved a total distance during the trial that was ~40–50% less than that of younger mice, regardless of genotype. In age-matched mice, there were no significant differences between distances achieved at exhaustion. 

While measures of distance and time to exhaustion are continuous variables and therefore tend to have greater variation in outcomes, the maximal speed achieved is a discrete variable since it is increased in stepwise increments. When maximal speeds were analyzed, a difference between genotypes was observed. Mice at 12 months of age were able to achieve a significantly lower maximal speed than mice at 3 months of age, regardless of genotype, indicating an age-related decline in performance in both groups, which was similar to that observed for both time to exhaustion and distance to exhaustion measures. However, a small but significant difference between *Wt* and *Taz*-HET mice was observed specifically at 12 months of age. Since the treadmill speed was increased in 3 cm/s increments, the maximum speed achieved by *Wt* mice was, on average, 49 cm/s, while the maximum speed achieved by *Taz*-HET mice was, on average, 46 cm/s, indicating that the performance of *Taz*-HET females peaked at a level lower than that of the *Wt* females.

### 3.9. Tafazzin Gene Expression and Cardiolipin Analysis

**Figure 9:**
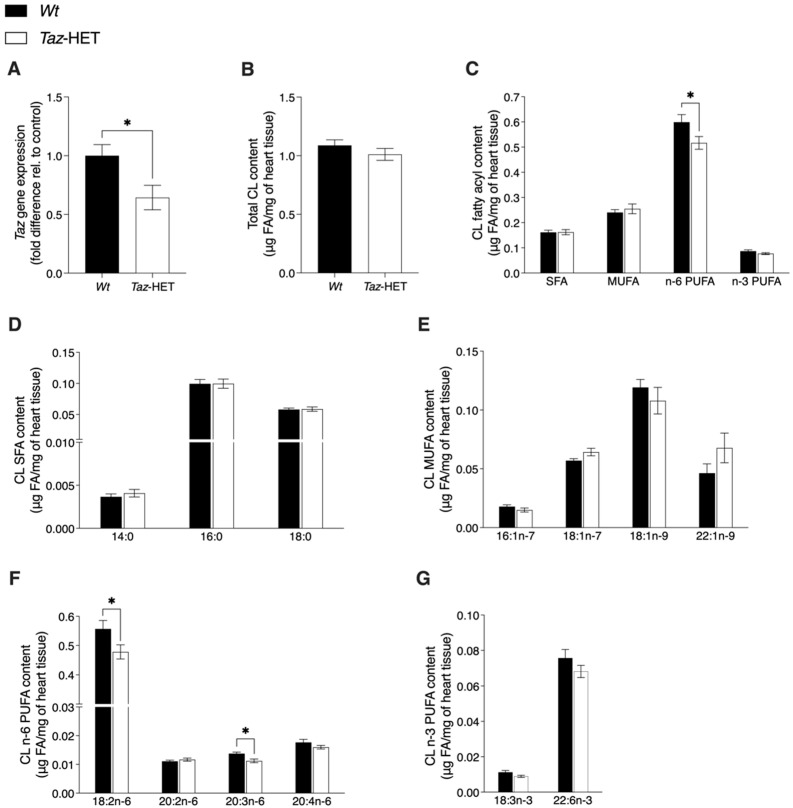
Tafazzin (*Taz*) gene expression and cardiolipin analysis in the cardiac tissue of 12-month-old mice. Tafazzin gene expression, normalized to β-actin, in the hearts of *Taz*-HET and control female mice (**A**). Total cardiolipin content (**B**), absolute content of major fatty acyl (FA) classes (i.e., saturates (SFA), monounsaturates (MUFA), n-6 polyunsaturated fatty acids (n-6 PUFAs), and n-3 PUFAs within cardiolipin (**C**), and cardiolipin absolute contents of specific FA species among SFA (**D**), MUFA (**E**), n-6 PUFA (**F**), and n-3 PUFA (**G**). Data are means ± SEM, *n* = 9–12, * *p* < 0.05.

Altered exercise performance in middle-aged *Taz*-HET females prompted us to investigate tafazzin expression and cardiolipin content in the hearts of mice at this timepoint. Tafazzin mRNA levels were ~36% lower in the hearts of *Taz*-HET females compared with *Wt* females (Figure 9A). Despite a lack of statistically significant differences in total cardiolipin content between *Taz*-HET and *Wt* females (Figure 9B), the total n-6 polyunsaturated fatty acid (PUFA) content was significantly lower in the 12-month-old *Taz*-HET females compared with age-matched *Wt* females (Figure 9C). Analysis of the abundance of specific fatty acyl species of cardiolipin revealed no differences in saturated fatty acids (SFA), monounsaturated fatty acids (MUFAs), and n-3 PUFAs (Figure 9C–E,G), although the n-6 PUFAs 18:2n-6 (linoleic acid) and 20:3n-6 (dihomo-γ-linolenic acid) were significantly lower by ~14% and ~18%, respectively, in the *Taz*-HET females (Figure 9F). Analysis of the relative abundance of fatty acyls in cardiac cardiolipin showed similar species-specific differences to those observed in absolute abundance comparisons and additionally showed a significant increase in MUFA content, particularly 18:1n-7 (vaccenic acid), in *Taz*-HET mice compared with *Wt* females (Supplementary Table S1).

## 4. Discussion

The molecular pathogenesis of BTHS involves cellular deficiencies in both L4-cardiolipin and total cardiolipin, together with an accumulation of monolysocardiolipin [14]. Female carriers are typically thought to be asymptomatic [14] and reportedly lack these hallmark biochemical abnormalities in tests of platelets and lymphocytes [27,28]. Additionally, carriers are also reported to lack perturbations in blood plasma measures of 3-methylglutaconic acid, cholesterol and cholesterol precursors, amino acids, and lactic acid that can occur in those with manifest disease [29,30,31]. Although a comprehensive clinical examination of carriers has not been performed, case reports also largely indicate an absence of female cardiac disease in the family history [32,33,34]. Moreover, assessment of cardiac function in mothers of BTHS patients typically reveals normal results through echocardiography [30,33,35,36] or electrocardiography [33]. 

We have recently published work reporting the characterization of male mice deficient in tafazzin (*Taz*-KO) [20]. In the present study, we assessed the characteristics of female mice that were heterozygous for a targeted disruption of the tafazzin gene. Whereas the male *Taz*-KO mice exhibited significant and sizeable reductions in body weights, major organ weights, adipose depot weights, and exercise capacity compared with male littermate control mice, the corresponding differences between *Taz*-HET female mice and their *Wt* female littermates were smaller. These results are largely consistent with the view that BTHS carriers are non-manifesting [14], since at both age points there were more similarities than differences between the two genotypes of female mice. Overall, changes that manifested with age in the *Taz*-HET females were subtle and unlikely to reach a threshold constituting clinically relevant pathology. Regardless, some differences were observed and are relevant for understanding the biological role of tafazzin in carriers.

*Taz*-HET mice were mostly indistinguishable from control littermates, as was expected based on reports from female carriers [14]. In instances where *Taz*-HET mice differed from their *Wt* counterparts, their phenotype recapitulated that of the *Taz*-KO males, but to a milder degree. Notable genotype-specific differences included lower body weights of 12-month-old *Taz*-HET mice compared with control females, in part due to lower weights of organs such as the brain and liver but more so due to smaller masses of select regional adipose depots. While the *Wt* mice had significantly reduced food intake with advancing age, this age-related change was lacking in the *Taz*-HET mice, despite their lower body weights. Systemic energy expenditure and basal substrate metabolism were comparable between the two groups at both age points and thus underwent similar age-related changes, while there were no notable differences in the movement or activity of the mice between genotypes at either age. Taken together, these data suggest some decreased efficiency of fuel use by female *Taz*-HET mice that may either manifest at an advanced age or compound as an accrued deficit over time. 

Neither genotype was overtly glucose intolerant or insulin resistant at 12-months of age, since mean blood glucose measures in both groups returned to baseline 2 h following glucose injection and both groups exhibited significant declines in blood glucose levels following insulin injection [37]. Differences between genotypes, however, were evident. Aged *Taz*-HET females exhibited an enhanced ability to maintain glucose homeostasis in the face of a glucose challenge, with a lower overall response during GTT. *Taz*-HET mice also exhibited a better response to insulin over the course of the ITT. In theory, these metabolic changes could offer health benefits with aging and may contribute to a lower risk of diabetes and obesity development [38], although this has not been studied yet in humans.

In the treadmill exercise capacity test, the maximum speed achieved, total running distance, and running time before exhaustion were the same between the *Taz*-HET and *Wt* mice at 3-months of age. At 12-months of age, however, a phenotypic difference in their performance became discernible, as the maximum speed achieved before exhaustion was slightly but significantly lower in the *Taz*-HET females. This finding of a difference is particularly interesting given that mitochondrial insufficiency and exercise intolerance are hallmarks of BTHS. To the best of our knowledge, this is the first study to identify a difference, albeit minor, in exercise capacity associated with heterozygous loss of tafazzin in female carriers. The largely protected phenotype of *Taz*-HET females could potentially be attributed to a relatively high degree of haplosufficiency of tafazzin or to a skewed X chromosome inactivation (XCI). 

Females have two X chromosomes, and, normally, one of the two is randomly chosen to be rendered transcriptionally inactive in each cell of the early embryo [39]. Throughout the cascading cell division that drives embryonic development, the inactivation of a specific X chromosome is permanent for all descendants of a cell [39]. Random XCI creates epigenetic cellular mosaicism in the tissues of females, which are composed of two populations of cells, with either the maternal or paternal chromosome as the active X [39]. In the framework of random XCI, one-half of the cells in a given tissue of *Taz*-HET mice should have the X chromosome with the mutant tafazzin gene active, and thus those cells should be deficient in the tafazzin protein. 

Although we did not directly analyze XCI patterns in the current study, we found that twelve-month-old *Taz*-HET females showed an ~1/3 reduction in cardiac tafazzin expression, suggesting that the X chromosome possessing the recombined knock-out tafazzin locus, missing exons 5–10, is likely active in a subset of cardiomyocytes of the mice. Since tafazzin has a well-established role in incorporating, specifically, linoleic acid chains into cardiolipin in mammalian hearts [40], thereby completing the transformation of nascent cardiolipin into its mature form specific to this tissue, it is noteworthy that the subtle decrease in tafazzin expression that we observed was associated with a correspondingly minor but significant and specific decline in cardiolipin linoleic acid content in the hearts of *Taz*-HET mice. Indeed, in BTHS patients [40], male tafazzin knock-down mice [5,41], and cardiomyocyte-specific tafazzin knockout mice [42], notable and specific reductions in the linoleic acid content of cardiac cardiolipin are observed. This decline is hypothesized to be causative of the hallmark exercise intolerance of BTHS [43,44], due to the associated decreases in concentration of the proteins of oxidative phosphorylation at the mitochondrial inner membrane that are enriched in L4-cardiolipin [45]. In the current work, the decrease in tafazzin expression, alterations in cardiolipin composition, and resulting exercise deficits are collectively minor in the *Taz*-HET female mice. This aligns with the notion that the *Taz*-HET model shares similarities with reports from human female carriers, where skewed XCI has been reported [32], but future studies should investigate this directly.

Studies of XCI status in human BTHS carriers suggest that skewed XCI is responsible for their asymptomatic status [32]. Ørstavik et al. analyzed the XCI status of peripheral blood cells from 16 obligate carriers of BTHS, none with known cardiac disease, and 148 female controls [32]. The majority of carriers possessed an almost completely skewed XCI pattern (>=95:5), since six of the sixteen carriers (i.e., 38%) expressed the X chromosome with the normal tafazzin gene in 95% or more of their blood cells, with 5% or less of their blood cells using the mutated X chromosome as the active X. This degree of imbalance in parental X chromosome activation was absent in the 148 controls. Extremely skewed XCI status was found in carriers at both early life and senior ages and was confirmed in skin fibroblasts and granulocytes collected from two of the carriers. Interestingly, random XCI inactivation (defined as ratios between 50:50 and 65:35) was observed in 19% of carriers (three out of sixteen) and was significantly lower than the 55% incidence observed in the control group. Notably, carriers with an extremely skewed status and with a random status were found in the same families. The remainder of carriers studied displayed moderately skewed (defined as 65:35 to 80:20) or skewed (defined as 80:20 to 95:5) XCI, and, thus, 81% of BTHS carriers possessed discernible levels of skewed XCI, favoring the expression of the normal tafazzin gene [32]. 

Levels of skewness can be clinically relevant in some disorders [46,47] and have been associated with the development of overt pathology in BTHS since predominant expression of the mutant X chromosome has been found in the two known cases of females with manifestations of clinical disease. The first case of BTHS in a female was reported in 2012 [35]. The infant presented with severe heart failure at 1 month of age, and her clinical course was characterized by recurrent episodes of severe acute heart failure, progressive skeletal myopathy, and cyclic neutropenia until she succumbed to a fatal septic shock at 3 years of age. Cytogenetic analysis performed on lymphocytes and skin fibroblasts determined that the girl had inherited a mutated maternal X chromosome with a large intragenic deletion for exons 1–5 of the tafazzin gene that would preclude the synthesis of a functional enzyme. Her paternal X chromosome was either structurally abnormal, in a ring conformation and missing the whole Xq28 region, or completely absent in cells. In effect, the female patient, like affected males with BTHS, was deficient in functional tafazzin and hence presented with clinical disease [35]. 

The second female BTHS patient, reported in 2016, was mildly affected by hypertrophic left-ventricular noncompaction and hypotonia [48]. She received a diagnosis of BTHS only after her younger male sibling presented with the classical phenotype. Genetic investigation of her peripheral blood cells revealed that the manifesting carrier daughter and her asymptomatic carrier mother had similar degrees of XCI skewness, but in opposite directions, where the daughter had the X chromosome with the mutant tafazzin gene as the transcriptionally active one in 97% of her cells, while her mother exhibited predominant activation of the normal X chromosome in 93% of her cells. Notably, the daughter had a random XCI pattern in epithelial cells collected from a buccal swab and urine specimens. The daughter had a normal karyotype, and it is unknown why the mutant X chromosome was preferentially activated in her blood cells and presumably other mesoderm-derived tissues [48]. Although further study is warranted, this demonstrates support for the notion that XCI patterns are a determinant of clinical phenotype in carriers with BTHS, where most but not all carriers exhibit skewness towards inactivation of the mutant X chromosome in a majority of their cells [32]. 

Although XCI was not analyzed in the present study, the relatively small differences observed suggest that *Taz*-HET mice undergo similar patterns to humans of skewed XCI, with a majority of mutant X chromosomes inactivated. Our findings of heterogeneity in performance responses and outcomes within genotype-age categories also suggest that, as with human carriers, female *Taz*-HET mice may experience variation in the degree of mutant XCI between individuals. As many as 19% of BTHS carriers possess random XCI [32], but whether this plays a causal role in clinical symptoms is yet unknown. Case reports occasionally produce results showing a carrier displaying mild cardiac symptoms, although these reports lack accompanying XCI analysis. For example, a woman who was the carrier of a de novo tafazzin mutation and the mother of a BTHS patient had mild trabeculations of the left ventricle as revealed by echocardiography, but her total cardiac function and electrocardiogram were normal [49]. In a different case, examination of the pedigree of a boy with BTHS possessing a newly documented tafazzin mutation revealed a female relative that was diagnosed with Wolff-Parkinson-White syndrome and affected by dilated cardiomyopathy [50]. This woman lost her son to dilated cardiomyopathy, and the presence of a tafazzin mutation and BTHS diagnosis was suspected but never confirmed due to a lack of sample acquisition prior to death. Nonetheless, genetic sequencing confirmed the woman as a carrier of the novel tafazzin mutation. Additionally, neutropenia in a carrier has also been observed [30], although this is the single exception to the majority of carriers that do not present with neutropenia [30,51]. 

It is possible that subtle, subclinical differences may exist between unaffected carriers and controls at the molecular level, even when there is apparent haplosufficiency. A study by Kuijpers et al. on annexin-V-based apoptosis in neutrophils from BTHS patients, carriers, and controls uncovered differences between the three subject groups [51]. Neutrophils from BTHS patients demonstrated avid binding to annexin-V. Although the neutrophils from carriers displayed a wide range in binding levels, they fell into an intermediate range between BTHS patients and healthy controls. None of the carriers had neutropenia, although the biological significance of the increased annexin-V binding is unknown, especially since Kuijpers et al. noted an absence of apoptosis in BTHS neutrophils despite their binding to annexin-V. Likewise, the underlying cause for increased annexin-V binding remains unclear, although cardiolipin-independent modifications to the cellular membranes have been suggested [51]. Future work should incorporate XCI analysis in order to examine the relative association between clinical symptoms of carrier women and the degree of tafazzin insufficiency.

We studied the *Taz*-HET mouse model at two different age points, focusing on cohorts corresponding to young (3-month-old) and middle-aged (12-month-old) timepoints [52], since age-related manifestations of subclinical and clinical features have been reported in carriers of other X-linked disorders, such as Duchenne muscular dystrophy [53,54,55], glucose-6-phosphate dehydrogenase deficiency [56,57], and X-linked adrenoleukodystrophy [17,58]. The importance of studying X-linked disorders across the lifespan is highlighted by the experience of discovery in X-linked adrenoleukodystrophy, where carriers were originally considered to be asymptomatic [59,60], leading to misdiagnoses with multiple sclerosis, hereditary spastic paraparesis, or fibromyalgia [61,62,63,64,65]. Although reports of manifesting carriers began to accumulate over decades [58,61,66,67,68,69], it was not until the first systematic study in 2014 on disease manifestations in carriers that it was established that more than 80% of women experience symptoms at ages 60 years or older [17]. Notably, this provided a sharp increase from the 18% of symptomatic women aged 40 years or younger [17]. 

Interestingly, in X-ALD, the significance of the XCI pattern for the symptomatic status remains uncertain, despite studies [17,70,71,72]. The effects of age on clinical outcomes have not been formally investigated in BTHS carriers, although extreme XCI skewness away from expression of the mutant tafazzin allele predominates in both children and seniors [32]. Our findings suggest that the *Taz*-HET mouse model can be used to further explore expected interactions between age, XCI status, and symptom penetrance and severity in human female carriers. 

## 5. Conclusions

Here, we have identified the development of subtle phenotypic differences between 12-month-old *Taz*-HET and *Wt* mice, which were nearly indistinguishable at 3 months of age. At 1 year of age, *Taz*-HET females have lower total body weights and enhanced glucose control compared with their *Wt* littermates. However, mild impairments in exercise capacity are also observed in the *Taz*-HET females at this age point, which are likely precipitated, in part, by lower tafazzin expression and linoleic acid content of cardiolipin in the hearts. While the subtle differences between the genotypes in this study corroborate the many reports of asymptomatic carriers of BTHS [27,29,30,31,32,33,34,35,36] and suggest that for the majority of BTHS carriers, abnormalities are likely to remain in a subclinical state, our findings of emergent differences at 12 months indicate that future work should examine later ages in both animals and humans. In particular, phenotypic differences may become more pronounced in model mice with advancing age, particularly at the threshold when aging progresses to senescence, which is typically observed around the 18-month milestone [52,73], and future studies should examine this directly.

## Data Availability

Data are available upon reasonable request to the authors.

## Supplementary Materials

The following supporting information can be downloaded at: https://www.mdpi.com/article/10.3390/biology12091238/s1, Table S1: Relative mass percentage of fatty acid species and major fatty acid classes within cardiolipin in the hearts of 12-month-old Taz-HET mice and Wt female littermates.

## References

[1] Pennington E.R., Funai K., Brown D.A., Shaikh S.R. (2019). The role of cardiolipin concentration and acyl chain composition on mitochondrial inner membrane molecular organization and function. Biochim. Biophys. Acta Mol. Cell Biol. Lipids.

[2] Bradley R.M., Stark K.D., Duncan R.E. (2016). Influence of tissue, diet, and enzymatic remodeling on cardiolipin fatty acyl profile. Mol. Nutr. Food Res..

[3] Han X., Yang K., Yang J., Cheng H., Gross R.W. (2006). Shotgun lipidomics of cardiolipin molecular species in lipid extracts of biological samples. J. Lipid Res..

[4] Schlame M., Towbin J.A., Heerdt P.M., Jehle R., DiMauro S., Blanck T.J. (2002). Deficiency of tetralinoleoyl-cardiolipin in Barth syndrome. Ann. Neurol..

[5] Acehan D., Vaz F., Houtkooper R.H., James J., Moore V., Tokunaga C., Kulik W., Wansapura J., Toth M.J., Strauss A. (2011). Cardiac and skeletal muscle defects in a mouse model of human Barth syndrome. J. Biol. Chem..

[6] Kennedy E.P., Weiss S.B. (1956). The function of cytidine coenzymes in the biosynthesis of phospholipides. J. Biol. Chem..

[7] Bione S., D’Adamo P., Maestrini E., Gedeon A.K., Bolhuis P.A., Toniolo D. (1996). A novel X-linked gene, G4.5. is responsible for Barth syndrome. Nat. Genet..

[8] Bradley R.M., Hashemi A., Aristizabal-Henao J.J., Stark K.D., Duncan R.E. (2022). PLAAT1 Exhibits Phosphatidylcholine: Monolysocardiolipin Transacylase Activity. Int. J. Mol. Sci..

[9] Ma B.J., Taylor W.A., Dolinsky V.W., Hatch G.M. (1999). Acylation of monolysocardiolipin in rat heart. J. Lipid Res..

[10] Taylor W.A., Hatch G.M. (2009). Identification of the human mitochondrial linoleoyl-coenzyme A monolysocardiolipin acyltransferase (MLCL AT-1). J. Biol. Chem..

[11] Taylor W.A., Mejia E.M., Mitchell R.W., Choy P.C., Sparagna G.C., Hatch G.M. (2012). Human trifunctional protein alpha links cardiolipin remodeling to beta-oxidation. PLoS ONE.

[12] Cao J., Liu Y., Lockwood J., Burn P., Shi Y. (2004). A novel cardiolipin-remodeling pathway revealed by a gene encoding an endoplasmic reticulum-associated acyl-CoA:lysocardiolipin acyltransferase (ALCAT1) in mouse. J. Biol. Chem..

[13] Zegallai H.M., Hatch G.M. (2021). Barth syndrome: Cardiolipin, cellular pathophysiology, management, and novel therapeutic targets. Mol. Cell. Biochem..

[14] Clarke S.L., Bowron A., Gonzalez I.L., Groves S.J., Newbury-Ecob R., Clayton N., Martin R.P., Tsai-Goodman B., Garratt V., Ashworth M. (2013). Barth syndrome. Orphanet J. Rare Dis..

[15] Rigaud C., Lebre A.-S., Touraine R., Beaupain B., Ottolenghi C., Chabli A., Ansquer H., Ozsahin H., Di Filippo S., De Lonlay P. (2013). Natural history of Barth syndrome: A national cohort study of 22 patients. Orphanet J. Rare Dis..

[16] Huhta J.C., Pomerance H.H., Barness E.G. (2005). Clinicopathologic conference: Barth Syndrome. Fetal Pediatr. Pathol..

[17] Engelen M., Barbier M., Dijkstra I.M., Schür R., de Bie R.M., Verhamme C., Dijkgraaf M.G., Aubourg P.A., Wanders R.J., van Geel B.M. (2014). X-linked adrenoleukodystrophy in women: A cross-sectional cohort study. Brain.

[18] Wang S., Li Y., Xu Y., Ma Q., Lin Z., Schlame M., Bezzerides V.J., Strathdee D., Pu W.T. (2020). AAV Gene Therapy Prevents and Reverses Heart Failure in a Murine Knockout Model of Barth Syndrome. Circ. Res..

[19] Ren M., Xu Y., Erdjument-Bromage H., Donelian A., Phoon C.K.L., Terada N., Strathdee D., Neubert T.A., Schlame M. (2019). Extramitochondrial cardiolipin suggests a novel function of mitochondria in spermatogenesis. J. Cell Biol..

[20] Tomczewski M.V., Chan J.Z., Campbell Z.E., Strathdee D., Duncan R.E. (2023). Phenotypic Characterization of Male Tafazzin-Knockout Mice at 3, 6, and 12 Months of Age. Biomedicines.

[21] Even P.C., Nadkarni N.A. (2012). Indirect calorimetry in laboratory mice and rats: Principles, practical considerations, interpretation and perspectives. Am. J. Physiol. Regul. Integr. Comp. Physiol..

[22] Lusk G. (1924). ANIMAL CALORIMETRY Twenty-Fourth Paper. Analysis of the oxidation of mixtures of carbohydrate and fat. J. Biol. Chem..

[23] Virtue S., Vidal-Puig A. (2021). GTTs and ITTs in mice: Simple tests, complex answers. Nat. Metab..

[24] Handschin C., Chin S., Li P., Liu F., Maratos-Flier E., LeBrasseur N.K., Yan Z., Spiegelman B.M. (2007). Skeletal Muscle Fiber-type Switching, Exercise Intolerance, and Myopathy in PGC-1α Muscle-specific Knock-out Animals. J. Biol. Chem..

[25] Claghorn G.C., Fonseca I.A.T., Thompson Z., Barber C., Garland T. (2016). Serotonin-mediated central fatigue underlies increased endurance capacity in mice from lines selectively bred for high voluntary wheel running. Physiol. Behav..

[26] Chan J.Z., Fernandes M.F., Steckel K.E., Bradley R.M., Hashemi A., Groh M.R., Sciaini G., Stark K.D., Duncan R.E. (2022). N-oleoylethanolamide treatment of lymphoblasts deficient in Tafazzin improves cell growth and mitochondrial morphology and dynamics. Sci. Rep..

[27] Schlame M., Kelley R.I., Feigenbaum A., Towbin J.A., Heerdt P.M., Schieble T., Wanders R.J., DiMauro S., Blanck T.J. (2003). Phospholipid abnormalities in children with Barth syndrome. J. Am. Coll. Cardiol..

[28] Houtkooper R.H., Rodenburg R.J., Thiels C., van Lenthe H., Stet F., Poll-The B.T., Stone J.E., Steward C.G., Wanders R.J., Smeitink J. (2009). Cardiolipin and monolysocardiolipin analysis in fibroblasts, lymphocytes, and tissues using high-performance liquid chromatography-mass spectrometry as a diagnostic test for Barth syndrome. Anal. Biochem..

[29] Vernon H.J., Sandlers Y., McClellan R., Kelley R.I. (2014). Clinical laboratory studies in Barth Syndrome. Mol. Genet. Metab..

[30] Płatek T., Sordyl M., Polus A., Olszanecka A., Kroczka S., Solnica B. (2023). Analysis of tafazzin and deoxyribonuclease 1 like 1 transcripts and X chromosome sequencing in the evaluation of the effect of mosaicism in the TAZ gene on phenotypes in a family affected by Barth syndrome. Mutat. Res..

[31] Johnston J., Kelley R.I., Feigenbaum A., Cox G.F., Iyer G.S., Funanage V.L., Proujansky R. (1997). Mutation characterization and genotype-phenotype correlation in Barth syndrome. Am. J. Hum. Genet..

[32] Orstavik K.H., Orstavik R.E., Naumova A.K., D’Adamo P., Gedeon A., Bolhuis P.A., Barth P.G., Toniolo D. (1998). X chromosome inactivation in carriers of Barth syndrome. Am. J. Hum. Genet..

[33] Bakšienė M., Benušienė E., Morkūnienė A., Ambrozaitytė L., Utkus A., Kučinskas V. (2016). A novel intronic splice site tafazzin gene mutation detected prenatally in a family with Barth syndrome. Balkan. J. Med. Genet..

[34] Barth P.G., Scholte H.R., Berden J.A., Van der Klei-Van Moorsel J.M., Luyt-Houwen I.E., Van’t Veer-Korthof E.T., Van der Harten J.J., Sobotka-Plojhar M.A. (1983). An X-linked mitochondrial disease affecting cardiac muscle, skeletal muscle and neutrophil leucocytes. J. Neurol. Sci..

[35] Cosson L., Toutain A., Simard G., Kulik W., Matyas G., Guichet A., Blasco H., Maakaroun-Vermesse Z., Vaillant M.C., Le Caignec C. (2012). Barth syndrome in a female patient. Mol. Genet. Metab..

[36] Ichida F., Tsubata S., Bowles K.R., Haneda N., Uese K., Miyawaki T., Dreyer W.J., Messina J., Li H., Bowles N.E. (2001). Novel gene mutations in patients with left ventricular noncompaction or Barth syndrome. Circulation.

[37] Alquier T., Poitout V. (2018). Considerations and guidelines for mouse metabolic phenotyping in diabetes research. Diabetologia.

[38] Brewer R.A., Gibbs V.K., Smith D.L. (2016). Targeting glucose metabolism for healthy aging. Nutr. Healthy Aging.

[39] Orstavik K.H. (2009). X chromosome inactivation in clinical practice. Hum. Genet..

[40] Schlame M., Ren M., Xu Y., Greenberg M.L., Haller I. (2005). Molecular symmetry in mitochondrial cardiolipins. Chem. Phys. Lipids.

[41] Soustek M.S., Falk D.J., Mah C.S., Toth M.J., Schlame M., Lewin A.S., Byrne B.J. (2011). Characterization of a transgenic short hairpin RNA-induced murine model of Tafazzin deficiency. Hum. Gene Ther..

[42] Zhu S., Chen Z.e., Zhu M., Shen Y., Leon L.J., Chi L., Spinozzi S., Tan C., Gu Y., Nguyen A. (2021). Cardiolipin Remodeling Defects Impair Mitochondrial Architecture and Function in a Murine Model of Barth Syndrome Cardiomyopathy. Circ. Heart Fail..

[43] Powers C., Huang Y., Strauss A., Khuchua Z. (2013). Diminished Exercise Capacity and Mitochondrial bc1 Complex Deficiency in Tafazzin-Knockdown Mice. Front. Physiol..

[44] Bashir A., Bohnert K.L., Reeds D.N., Peterson L.R., Bittel A.J., de Las Fuentes L., Pacak C.A., Byrne B.J., Cade W.T. (2017). Impaired cardiac and skeletal muscle bioenergetics in children, adolescents, and young adults with Barth syndrome. Physiol. Rep..

[45] Xu Y., Phoon C.K.L., Ren M., Schlame M. (2022). A simple mechanistic explanation for Barth syndrome and cardiolipin remodeling. J. Inherit. Metab. Dis..

[46] Ozcelik T. (2008). X chromosome inactivation and female predisposition to autoimmunity. Clin. Rev. Allergy Immunol..

[47] Ostan R., Monti D., Gueresi P., Bussolotto M., Franceschi C., Baggio G. (2016). Gender, aging and longevity in humans: An update of an intriguing/neglected scenario paving the way to a gender-specific medicine. Clin. Sci..

[48] Avdjieva-Tzavella D.M., Todorova A.P., Kathom H.M., Ivanova M.B., Yordanova I.T., Todorov T.P., Litvinenko I.O., Dasheva-Dimitrova A.T., Tincheva R.S. (2016). Barth Syndrome in Male and Female Siblings Caused by a Novel Mutation in the Taz Gene. Genet. Couns..

[49] Bachou T., Giannakopoulos A., Trapali C., Vazeou A., Kattamis A. (2009). A novel mutation in the G4.5 (TAZ) gene in a Greek patient with Barth syndrome. Blood Cells Mol. Dis..

[50] Brión M., de Castro López M.J., Santori M., Pérez Muñuzuri A., López Abel B., Baña Souto A.M., Martínez Soto M.I., Couce M.L. (2016). Prospective and Retrospective Diagnosis of Barth Syndrome Aided by Next-Generation Sequencing. Am. J. Clin. Pathol..

[51] Kuijpers T.W., Maianski N.A., Tool A.T., Becker K., Plecko B., Valianpour F., Wanders R.J., Pereira R., Van Hove J., Verhoeven A.J. (2004). Neutrophils in Barth syndrome (BTHS) avidly bind annexin-V in the absence of apoptosis. Blood.

[52] Dutta S., Sengupta P. (2016). Men and mice: Relating their ages. Life Sci..

[53] Ishizaki M., Kobayashi M., Adachi K., Matsumura T., Kimura E. (2018). Female dystrophinopathy: Review of current literature. Neuromuscul. Disord..

[54] Politano L., Nigro V., Nigro G., Petretta V.R., Passamano L., Papparella S., Di Somma S., Comi L.I. (1996). Development of cardiomyopathy in female carriers of Duchenne and Becker muscular dystrophies. Jama.

[55] Adachi K., Kawai H., Saito M., Naruo T., Kimura C., Mine H., Inui T., Kashiwagi S., Akaike M. (1997). Plasma levels of brain natriuretic peptide as an index for evaluation of cardiac function in female gene carriers of Duchenne muscular dystrophy. Intern. Med..

[56] Au W.Y., Ma E.S., Lam V.M., Chan J.L., Pang A., Kwong Y.L. (2004). Glucose 6-phosphate dehydrogenase (G6PD) deficiency in elderly Chinese women heterozygous for G6PD variants. Am. J. Med. Genet. A.

[57] Au W.Y., Lam V., Pang A., Lee W.M., Chan J.L., Song Y.Q., Ma E.S., Kwong Y.L. (2006). Glucose-6-phosphate dehydrogenase deficiency in female octogenarians, nanogenarians, and centenarians. J. Gerontol. A Biol. Sci. Med. Sci..

[58] Schmidt S., Träber F., Block W., Keller E., Pohl C., von Oertzen J., Schild H., Schlegel U., Klockgether T. (2001). Phenotype assignment in symptomatic female carriers of X-linked adrenoleukodystrophy. J. Neurol..

[59] Davis L.E., Snyder R.D., Orth D.N., Nicholson W.E., Kornfeld M., Seelinger D.F. (1979). Adrenoleukodystrophy and adrenomyeloneuropathy associated with partial adrenal insufficiency in three generations of a kindred. Am. J. Med..

[60] Heffungs W., Hameister H., Ropers H.H. (1980). Addison disease and cerebral sclerosis in an apparently heterozygous girl: Evidence for inactivation of the adrenoleukodystrophy locus. Clin. Genet..

[61] Jangouk P., Zackowski K.M., Naidu S., Raymond G.V. (2012). Adrenoleukodystrophy in female heterozygotes: Underrecognized and undertreated. Mol. Genet. Metab..

[62] Moser H.W., Moser A.B., Naidu S., Bergin A. (1991). Clinical aspects of adrenoleukodystrophy and adrenomyeloneuropathy. Dev. Neurosci..

[63] Costello D.J., Eichler A.F., Eichler F.S. (2009). Leukodystrophies: Classification, diagnosis, and treatment. Neurologist.

[64] Dooley J.M., Wright B.A. (1985). Adrenoleukodystrophy mimicking multiple sclerosis. Can. J. Neurol. Sci..

[65] Stöckler S., Millner M., Molzer B., Ebner F., Körner E., Moser H.W. (1993). Multiple sclerosis-like syndrome in a woman heterozygous for adrenoleukodystrophy. Eur. Neurol..

[66] O’Neill B.P., Moser H.W., Saxena K.M., Marmion L.C. (1984). Adrenoleukodystrophy: Clinical and biochemical manifestations in carriers. Neurology.

[67] van Geel B.M., Koelman J.H., Barth P.G., Ongerboer de Visser B.W. (1996). Peripheral nerve abnormalities in adrenomyeloneuropathy: A clinical and electrodiagnostic study. Neurology.

[68] Horn M.A., Retterstøl L., Abdelnoor M., Skjeldal O.H., Tallaksen C.M. (2013). Adrenoleukodystrophy in Norway: High rate of de novo mutations and age-dependent penetrance. Pediatr. Neurol..

[69] Jung H.H., Wimplinger I., Jung S., Landau K., Gal A., Heppner F.L. (2007). Phenotypes of female adrenoleukodystrophy. Neurology.

[70] Maier E.M., Kammerer S., Muntau A.C., Wichers M., Braun A., Roscher A.A. (2002). Symptoms in carriers of adrenoleukodystrophy relate to skewed X inactivation. Ann. Neurol..

[71] Migeon B.R., Moser H.W., Moser A.B., Axelman J., Sillence D., Norum R.A. (1981). Adrenoleukodystrophy: Evidence for X linkage, inactivation, and selection favoring the mutant allele in heterozygous cells. Proc. Natl. Acad. Sci. USA.

[72] Watkiss E., Webb T., Bundey S. (1993). Is skewed X inactivation responsible for symptoms in female carriers for adrenoleucodystrophy?. J. Med. Genet..

[73] Flurkey K., Currer J.M., Harrison D.E., Fox J.G., Davisson M.T., Quimby F.W., Barthold S.W., Newcomer C.E., Smith A.L. (2007). Chapter 20—Mouse Models in Aging Research. the Mouse in Biomedical Research.

