# Supplementary material for: Phenotypic Characterization of Female Carrier Mice Heterozygous for Tafazzin Deletion
Source: Biology (Basel). 2023 Sep 14;12(9):1238. doi: 10.3390/biology12091238 (PMC10525480; doi:10.3390/biology12091238)
Supplement: Supplementary file 1 [file biology-12-01238-s001.zip › biology-2581339-supplementary.pdf]

Supplementary Table S1. Relative mass percentage of fatty acid species and major fatty acid classes within cardiolipin in the hearts of 12-month-old *Taz*-HET mice and *Wt* female littermates.

Fatty acid	*Wt* females (*n* = 9)	*Taz*-HET females (*n* = 12)	P value
14:0	0.35 ± 0.02	0.38 ± 0.03	0.5550
16:0	8.91 ± 0.46	9.55 ± 0.45	0.3390
18:0	5.23 ± 0.12	5.69 ± 0.21	0.1021
SFAs	14.49 ± 0.50	15.61 ± 0.54	0.1577
16:1n-7	1.59 ± 0.11	1.42 ± 0.12	0.3226
**18:1n-7**	**5.16 ± 0.14**	**6.22 ± 0.22**	**0.0014**
18:1n-9	10.74 ± 0.49	10.26 ± 0.70	0.6027
22:1n-9	4.13 ± 0.64	6.36 ± 1.02	0.1052
**MUFAs**	**21.64 ± 0.63**	**24.26 ± 0.94**	**0.0443**
**18:2n-6**	**49.92 ± 0.90**	**46.08 ± 1.15**	**0.0221**
20:2n-6	1.01 ± 0.04	1.14 ± 0.04	0.0613
**20:3n-6**	**1.22 ± 0.03**	**1.08 ± 0.04**	**0.0173**
20:4n-6	1.57 ± 0.03	1.57 ± 0.06	0.9918
**n-6 PUFAs**	**53.72 ± 0.88**	**49.87 ± 1.16**	**0.0216**
18:3n-3	1.00 ± 0.08	0.85 ± 0.04	0.0802
22:6n-3	6.80 ± 0.30	6.63 ± 0.30	0.7034
n-3 PUFAs	7.80 ± 0.29	7.48 ± 0.28	0.4442
Total FA (μg/mg heart)	1.09 ± 0.05	1.01 ± 0.05	0.2984

Values are percentages of total fatty acids (FA) within cardiolipin, and expressed as mean ± S.E.M. Values reaching the statistical threshold of $P < 0.05$ are bolded. SFA: saturated fatty acids; MUFA: monounsaturated fatty acids; PUFAs: polyunsaturated fatty acids.
